# The Potential Role of Phytonutrients Flavonoids Influencing Gut Microbiota in the Prophylaxis and Treatment of Inflammatory Bowel Disease

**DOI:** 10.3389/fnut.2021.798038

**Published:** 2021-12-14

**Authors:** Lina Wang, Mengxue Gao, Guangbo Kang, He Huang

**Affiliations:** ^1^Department of Biochemical Engineering, School of Chemical Engineering and Technology, Tianjin University, Tianjin, China; ^2^Frontiers Science Center for Synthetic Biology and Key Laboratory of Systems Bioengineering, Ministry of Education, Tianjin University, Tianjin, China

**Keywords:** phytonutrients, flavonoids, gut microbiota, IBD, gut homeostasis

## Abstract

Inflammatory bowel disease (IBD), characterized by the chronic inflammation of the gastrointestinal tract, is comprised of two idiopathic chronic intestinal inflammatory diseases. As the incidence of IBD increases, so does the need for safe and effective treatments. Trillions of microorganisms are colonized in the mammalian intestine, coevolve with the host in a symbiotic relationship. Gut microbiota has been reported to be involved in the pathophysiology of IBD. In this regard, phytonutrients flavonoids have received increasing attention for their anti-oxidant and anti-inflammatory activities. In this review, we address recent advances in the interactions among flavonoids, gut microbiota, and IBD. Moreover, their possible potential mechanisms of action in IBD have been discussed. We conclude that there is a complex interaction between flavonoids and gut microbiota. It is expected that flavonoids can change or reshape the gut microbiota to provide important considerations for developing treatments for IBD.

## Introduction

In recent years, some studies have investigated complex and chronic diseases, including Inflammatory bowel disease (IBD), and focused on their association with the gut microbiota in the intestines. The incidence of IBD has persistently increased with over 3.5 million affected people and has become a global epidemic disease threatening human health (1, 2). Two major defined forms of IBD include ulcerative colitis (UC) and Crohn's disease (CD), characterized by chronic progressive inflammation, which affects the whole gastrointestinal tract and colon mucosa, respectively, leading to an increased risk of colon cancer. There has been substantial research undertaken on the cause of IBD, such as genetic interaction between the human gut microbiome and mucosal immune system, as well as changes in environmental factors (3). There is an intricate interrelationship between the IBD and gut microbiota. It has been reported that gut microbes consist of about 100 trillion microorganisms. The ratio of the number of gut microbes to human cells is close to 1:1, covering more than 1,100 species known to the human body, and the genes displayed are 100 times that of the human genome (4–6). This implies that the gut microbiota is a key component in the human body, playing important roles in the regulation of intestinal permeability, education of host immune responses, metabolism of dietary nutrients, and host's protection against invading pathogens (7, 8). With the increase in the availability of multi-omics approaches, the gradually-revealed mechanism of interaction between gut microbiota and the host has been thought to be distinct in healthy individuals than the IBD patients. The development of various large-scale microbiome projects, such as metagenomics of human intestinal tract (MetaHIT) (5), human microbiome project (HMP), and integrative human microbiome project (iHMP) (9, 10) reveals that the composition, diversity, and stability of gut microbiota are associated with the normal physiological functions and occurrence of diseases (11). The disorders in intestinal microbiota result in perturbation in the intestinal homeostasis and eventually contribute to the IBD.

Diet is a major contributor in shaping the composition and function of human intestinal microbiota by directly or indirectly affects. The complex interactions between diet and intestinal microbiota determines the beneficial or harmful effects on human health, which may be due to the immunomodulatory effects of the improved microbiota, downstream effects on host gene expression, or changes in the metabolite landscape produced by the microbiota (12). The increasing numbers of epidemiological studies have pointed toward the beneficial effects of phytonutrients to decrease the incidence of chronic diseases, including cardiovascular diseases, neurodegenerative diseases, and cancer (13, 14). Flavonoids are present as secondary metabolites in various fruits and vegetables in their different parts, representing a large representative family of the phytonutrients compounds with anti-oxidants and anti-microbial properties. Flavonoids are supplied as nutrition and food additives in functional dietary supplements because of their beneficial effects on human health (15, 16). Some studies have simultaneously demonstrated the beneficial effects of flavonoids on host energy metabolism by regulating the intestinal microbiota and suggested that the diversity and abundance of the intestinal microbiota are associated with the consumption of flavonoids (17–19). At present, flavonoids are considered to be vital for the treatment of acute or chronic intestinal inflammation through multiple mechanisms, including resistance to oxidative stress, protection of intestinal epithelial barrier, and immunomodulation (20). In this review, we summarized the classification and structural characteristics of phytonutrient flavonoids based on previous studies. We also described the latest advances in our understanding of how flavonoids and gut microbiota act independently or synergistically to influence IBD.

## Phytonutrients Flavonoids

Natural compounds isolated from plants hold a large reservoir of important bioactive molecules for drugs' discovery. Phytonutrients, also called nutritive phytochemicals, are secondary metabolites accumulated in the different parts of plants (21, 22). The bioactive compounds have potential to be applied as therapeutic mediators in the treatment of diseases. Despite the fact that most of the phytonutrients are non-essential for humans unlike vitamins or other essential mineral micronutrients, they contribute to a significant protection against chronic diseases (23, 24). In traditional medical practices, the phytonutrients have been widely used not only in dietary supplements but also as therapeutic agents for the enhancement of immunity and prevention of diseases (25). On the basis of their unique properties and various structures, the phytonutrients are classified into phenolic acids, flavonoids, carotenoids, tocochromanols, and curcuminoids (26).

**Figure 1:**
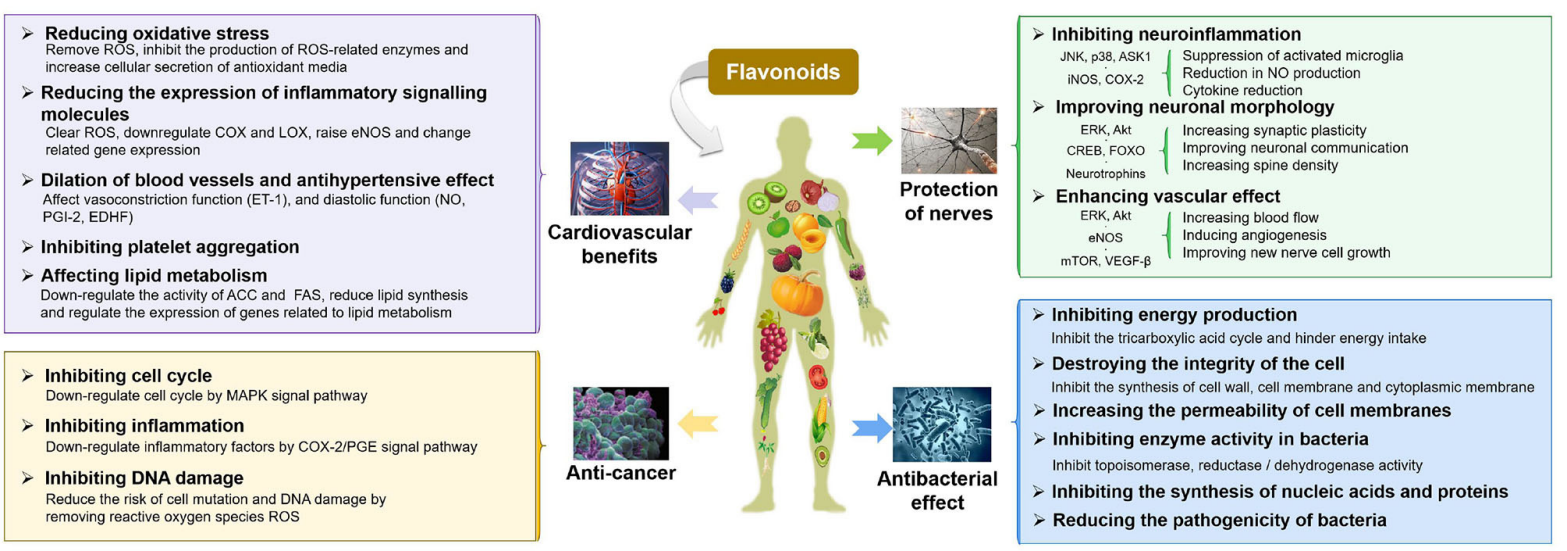
A summary of physiological functions and their corresponding mechanisms of flavonoids.

Flavonoids, a large family of phytonutrients, are one of the most biologically important poly-phenolic compounds, which occur ubiquitously in plant-based diets or medicinal plants, and comprise of a large group of unique compounds with oxygen-containing heterocyclic framework. The previous studies on flavonoids were focused on their curative effect because of their natural anti-oxidants, anti-carcinogenic (27, 28), anti-atherogenic (29), anti-ulcer (30), anti-thrombotic (31, 32), anti-inflammatory (33, 34), anti-allergenic (35), anti-coagulant (36, 37), immune modulating (34), anti-microbial (38, 39), vaso-dilatory (40, 41), and analgesic activities, which are summarized in Figure 1 (13, 42–44). The neuro-protective effects of flavonoids have been demonstrated in epidemiological studies to change the trajectories of degenerative diseases and improve the cognitive functions (45–49). Similarly, there are some evidences demonstrating the beneficial effect of flavonoids on cardiovascular and cerebrovascular health, either directly or indirectly by acting on the signaling molecules, biomarkers of oxidative stress, platelet function, and lipid metabolism, etc. (50–54). The anti-microbial properties of flavonoids have been extensively studied. The anti-bacterial mechanisms of flavonoids may include: damaging the structure of the cell membrane, inhibiting the synthesis of proteins, nucleic acids, or cell wall's components, and decreasing the production of energy (55–58). A growing body of evidence suggested flavonoids could inhibit carcinogenesis and the development of tumors by multiple mechanisms, such as showing anti-inflammatory effects (suppressing the expression of lipoxygenase, lipoxygenase, and inducible nitric oxide synthase, etc.) (59, 60). Dietary flavonoids were reported to have good anti-oxidant properties as scavengers of ROS and RNS, which can strongly inhibit the progression of inflammation, including inhibiting the release of inflammatory mediators and regulating inflammation-related signal pathways (NF-κB, AP-1, PPAR, Nrf2, and MAPK, etc.) (61). This review will focus on the beneficial effects of flavonoids on IBD, including protection of the intestinal epithelial cell barrier function, prevention of oxidative stress, and immunomodulatory properties, which will be discussed in detail in the following sections.

**Figure 2:**
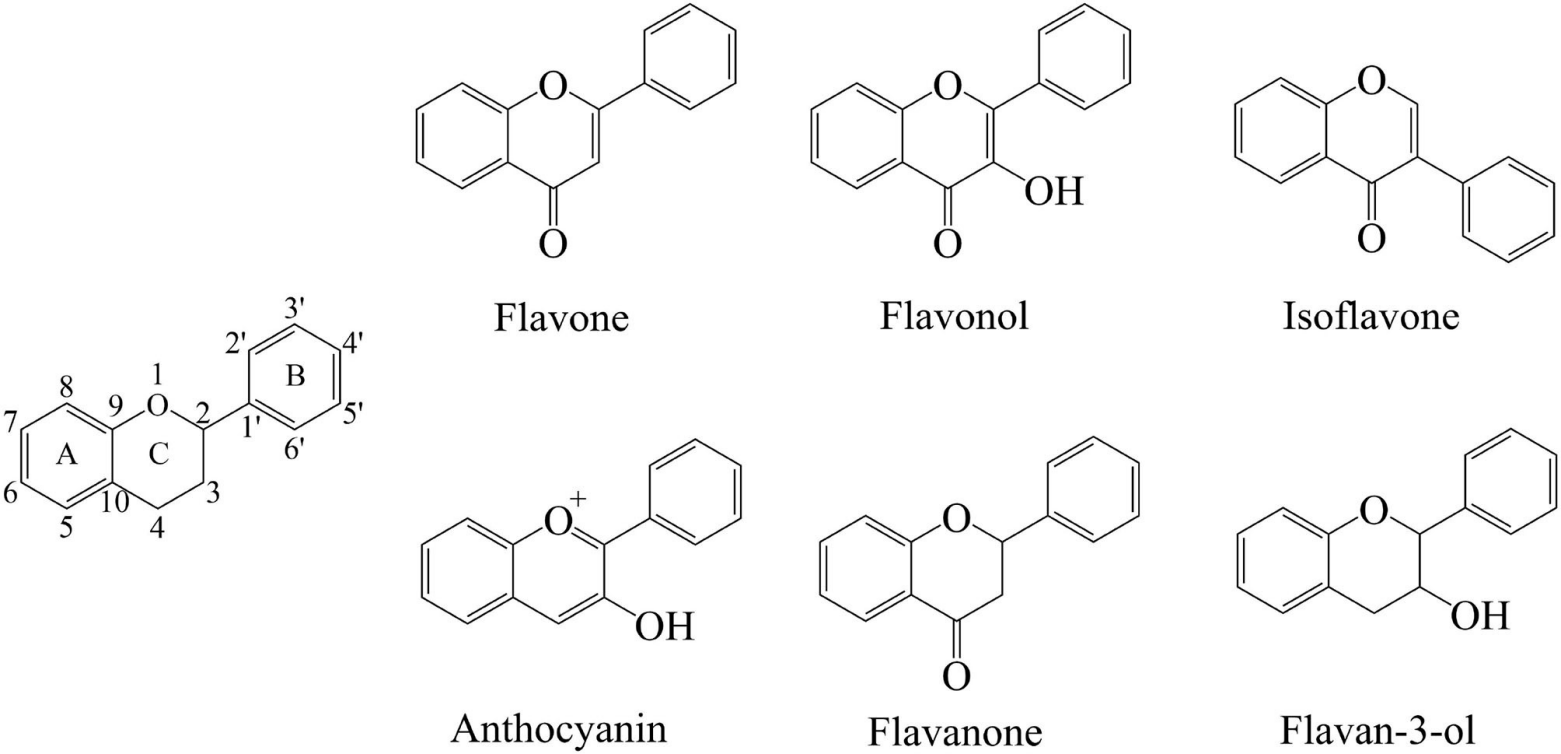
Skeleton structure diagram of flavonoids.

The flavonoids are a group of organic compounds with diverse chemical structures, characterized with structural backbone C6–C3–C6 called 2-phenylbenzo-pyran, composed of two benzene rings (A and B) and a oxygen-containing six-membered hetero-cycles. In the presence of the basic skeleton of flavonoids, many types of compounds are formed due to different chemical reactions, including glycosylation, hydroxylation, methoxylation, and prenylation. Flavonoids are classified into several different types, including flavonols, flavones, isoflavones, flavan-3-ols, flavanones, and anthocyanins. These compounds sometimes also occur as glycosides, methylated derivatives, or aglycones (Figure 2) (13, 62, 63).

Flavones consist of a 2-phenyl-benzo-γ-pyrone skeleton, which is comprised of a heterocyclic pyrone ring linked with two phenyl rings. The structure of flavones appears very similar to that of the flavanones, with the major differences in the formation of linear pyran rings depending on the unsaturated C2–C3 bonds. The flavonoids usually occur in conjugated forms as glycosides, and the flavones are generally found with O-glycosides at the C-3 or C-7 position, where the O-glycosidic linkage connects the sugar group with aglycone. On the other hand, the C-glycosides in flavones have received less attention, the C-glycosidic linkage is a key structural component (64, 65). Flavones are most commonly found in herbs such as parsley and celery, but they are also widely distributed in grains such as wheat, rye, barley, oats, sorghum, and millet. The most prevalent compounds of flavones include apigenin, baicalein, and luteolin. Their glycoside form compounds are vitexin, isovitexin, baicalin, luteolin 7-O-glucuronide, respectively. Apigenin is found in many vegetables and herbal spices, including parsley, celery, basil, chamomile, coriander, and oregano. Baicalein is a traditional Chinese herbal flavonoid found in the roots of *Scutellaria baicalensis* Georgi. Luteolin exists in a variety of plants, and these plants have a higher content of whole-leaf green orchid, pepper, wild chrysanthemum, honeysuckle, and perilla.

Flavonols have typical structural features within the plane of 3-hydroxyflavone base, which are distinguished according to the modifications in their different hydroxyl groups in benzene rings (62). Green tea, red grapes, onions, especially red onions, leafy vegetables, and berries are the main sources of flavonols. The highest content of flavonols has been observed in berries and onions (66). Distinguished from flavones, the flavonols have one hydroxyl group at C-3 position, which increases their instability but makes them prone to have O-glycosidic linkages in the form of O-glycosides. In addition, the flavonols are also found as O-glycosides at C-7 or other positions having hydroxyl moieties (67). The most common flavonols include quercetin, kaempferol, and myricetin, most of which form glycosidic combinations. Consequently, the glycosylation of these compounds result in enhancing their stability (68). Quercetin is widely found in various foods, including apples, black tea, berries, capers, red wine, and onions. Among them, onions have the highest content of quercetin. Kaempferol is widely distributed in different plant genera such as delphinium, camellia, barberry, tangerine peel. Myricetin is widely found in many natural plants including bayberry.

Isoflavones have a unique structure having B-ring at C-3 rather than at C-2, which is different from the flavones. Not only that, the isoflavones also structurally resemble endogenous estrogens and have estrogenic activity by binding estrogen receptor (69). The primary natural source of isoflavones is soybean, containing various types of compounds, which are classified according to aglycones or modification by other functional groups, such as methoxylation and hydroxylation (70). Genistein, daidzein, and glycitein are the main representative compounds of isoflavones, which belong to soy phytoestrogens. Genistein is a kind of phytoestrogens derived from soybeans, mainly found in the rhizomes of the leguminous plant Genistein. Daidzein is mainly found in the root of the legume *Pueraria lobata*, the whole legume red clover, and the whole alfalfa. Notably, the equol, which is a metabolite of daidzein, is not a natural plant compound but sometimes ranked among the isoflavones (71).

Anthocyanins contain conjugated double bonds that contribute to chromophore. Moreover, the hydroxylation and methylation also determine their color phenotypes and stability with a wide range of structures varying from simple to complex compounds (72). As water-soluble pigments, the anthocyanins are usually present in cell vacuoles of the plant tissues and tend to occur mainly as glycosides. The most common sugars bonded to them are polysaccharide or monosaccharide, including glucose, rutinose, rhamnose, and xylose (73). Anthocyanins are particularly present in red fruits, some cereals, and root vegetables, such as grapes, purple grapes, blackberries, strawberries, and raspberries. The most common identified anthocyanins include cyanidin, malvidin, petunidin, pelargonidin, delphinidin, and peonidin.

Distinguished from flavones, the flavanones have saturated C2–C3 bonds with non-linear structure and higher bioavailability. The common flavanones include naringenin, eriodictyol, hesperetin, and sakutanetin, which can form various glycosides by the glycosylation of glucose and rhamnose. Hesperidin and naringin are the representative glycosides abundant in citrus having beneficial effects on gastrointestinal health (63, 66).

Flavan-3-ols, also called flavanols, have a single hydroxyl group at C-3 position instead of double bond, and lack the carbonyl group at C4 position. Moreover, the structures of these compounds comprise of a wide range of compounds ranging from simple monomers to condensed tannins, including (+)-Catechin, (–)-Epicatechin, (–)-Epicatechin-3-gallate, gallocatechin, epigallocatechin, and epigallocatechin gallate (74).

## The Complex Interaction Between Flavonoids and Gut Microbiota

The ingested flavonoids, such as phytonutrients, indirectly provide energy and key elements in humans with health-promoting effects, where the gut microbiota serves a pivotal role. The gut microbiota comprises of microorganisms residing in the human gastrointestinal tract with the total number of more than 100 trillion cells (75). Its composition varies along the digestive tract and remains plastic according to the lifestyle as well as nutritional status of host throughout the life. The gut microbiota defines the gastrointestinal functional ecology by absorption, digestion, metabolism, and excretion of flavonoids, leading to the regulation of host immune response and behavior with long-term effects (17). Moreover, the changes in the composition of gut microbiota also introduce perturbation to bioavailability and biological effect of flavonoids. Consequently, the flavonoids regulate gut microbiota with varying levels of efficacy, including the stimulation of commensal and beneficial microbiota or the inhibition of the colonization of enteric pathogens (19, 76). It is therefore crucial to decipher the reciprocal correlation between flavonoids and gut microbiota.

### Regulation of Gut Microbiota by Flavonoids

Natural flavonoids can regulate the gut microbiota from the aspects of type and amount and ameliorate dysbiosis or maintain intestinal homeostasis, thereby maintaining the host gut health. Besides, the different types of flavonoids exhibit different efficacy in boosting or suppressing the growth of gut microbiota. Recent evidence suggests that the flavonoids from tart cherries substantially increase the specific *Bacteroides* and *Collinsella*. Furthermore, an *in vivo* study showed that gut microbiota composition changed with the consumption of tart cherries (77). In this case, the tart cherries flavonoids, such as cyanidin-glycosylrutinoside and quercetin-rutinoside, showed its prebiotic effects. In other examples, *in vitro* digestion models, simulating anaerobic human fecal fermentation, showed that Green tea, rich in flavones and flavonols, showed multiple health-beneficial properties by increasing the populations of *Lactobacilli* spp. and *Bifidobacteria* spp. (78). The cranberry or its extracts increased the abundance of *Akkermansia* spp. and inhibited *Bacteroides* and *Bifidobacteria*, reducing the intestinal inflammation (79). Three plant flavonoids, including quercetin, catechin, and puerarin, were compared in a study for their regulatory functions on gut microbiota *in vitro*. Experimental results suggested their different capacities to regulate the abundance of *Bifidobacterium* spp. Moreover, the aglycones rather than glycosides were shown to influence the growth of certain species. In detail, the quercetin stimulated *Firmicutes, Proteobacteria*, and *Actinobacteria*, while puerarin increased *Fusobacteria* and *Proteobacteria*, as the consequence of increasing the richness and diversity of microbial communities (80).

Many studies have reported the effects of flavonoids on the gut microbiota. *In vitro* models proved that the flavonols, such as quercetin, had the following prebiotic actions: increasing the abundance of *Bifidobacterium adolescentis*; anti-inflammatory activity by producing NO inhibitors in murine macrophage-like RAW264 cells (81); inhibiting gut pathogen *Salmonella typhimurium*'s growth and adhesion to human gut cell line; enhancing the proliferation and adhesion of probiotic *Lactobacillus rhamnosus* (82). Kaempferol was reported to suppress the vacuolating cytotoxin A and cytotoxin-associated gene A translocation of *Helicobacter pylori* in the AGS cells model (83). Baicalein showed the similar effects (84). *In vivo* experiments confirmed that the oral quercetin treatment in high-fat diet (HFD)-fed mice suppressed the abundance of *Verrucomicrobia* and increased that of *Actinobacteria, Cyanobacteria*, and *Firmicutes*, as well as the diversity of microbiota diversity (85–87). Baicalein regulated the composition of gut microbiota via its influence on the abundance of beneficial and pathogenic bacteria (88). The treatment of genistein the abundance among the *Prevotella* and *Akkermansia* genera, particularly *Prevotella copri* and *Akkermansia muciniphila* (89). Other special *in vivo* models, such as the APP/PS1 transgenic mice, found that under low vitamin D supplementation, quercetin elevated the diversity of gut microbiota including *Facklamia* and *Aerococcus*, improving the cognitive function (90). In female perinatal non-obese diabetic (NOD) mice, the exposure of genistein showed an increase in the level of *Enterobacteriales*, suggesting a pro-inflammatory response (91). In other experimental settings, the flavanones in *Citrus* were investigated for their interference with quorum sensing (QS), which showed reduction in the levels of QS signal mediated by acyl homoserine lactone (AHL) with the down-regulation of relative genes, and inhibition of biofilm formation as well as bacterium swimming and swarming motility (92). As for the flavan-3-ols, they changed the gut micro-ecology by affecting the adhesion of Lactobacilli strains to intestinal epithelial cells (93, 94). Anthocyanidin showed anti-bacterial activity by either killing the pathogenic bacteria or inhibiting their growth, such as *Bacillus subtilis, Staphylococcus aureus, Enterococcus faecalis*, and *Pseudomonas aeruginosa* (95). The regulation of gut microbiota by flavonoids is systematically summarized in Table 1. Previous studies were focused on the effects of the monomeric component of flavonoids on the specified strain of gut microorganisms. With the advancement in the development of metagenomics, metabolomics, proteomics, and transcriptomics, more research could focus on the in-depth regulatory mechanisms of flavonoids on gut microbiota.

**Table 1:** The regulatory effect of flavonoids on the gut microbiota.

**Compounds**	**Model/Material**	**Dosage form**	**Major findings**	**References**
**Flavonols**				
Quercetin	HFD Ldlr^−/−^ C57BL/6 mice	Oral administration of quercetin (100 μg day^−1^) supplement diets	Oral quercetin treatment suppressed the abundance of *Verrocomicrobia* and increased the abundances of *Actinobacteria, Cyanobacteria*, and *Firmicutes*, as well as microbiome diversity	(86)
	HFD male C57BL/6J mice divided into gut microbiota donor and receiver	Supplement diets with aglycone quercetin (0.05% wt/wt) at a dosage roughly equal to 80 mg kg^−1^ day^−1^	The notable increase of *Verrucomicrobia phylum, Verrucomicrobiae class*, and *Akkermansia genu* proved the prebiotic capacity of quercetin in gut microbiota transferring from donor to receiver mice model	(87)
	High-fat sucrose diet (HFS) Wistar rats	Feeding quercetin (30 mg/kg^−1^ day^−1^) as a supplement	Quercetin supplementation inhibited the growth of bacterial species previously associated with diet-induced obesity (*Erysipelotrichaceae, Bacillus, Eubacterium cylindroides*)	(85)
	Male APP/PS1 transgenic mice (B6C3F1)	Supplement with 0.08% quercetin roughly equal to 120 mg kg^−1^ d^−1^ in modified AIN-93G diet	Under low vitamin D status, quercetin elevated gut microbial diversity (including *Facklamia* and *Aerococcus*), and improved cognitive function	(90)
	*Pseudomonas aeruginosa* strain PAO1	PAO1 was cultured with quercetin (16 μg/ml)	Quercetin inhibited *Pseudomonas aeruginosa* by the factors of quorum sensing, biofilm formation, and virulence	(96)
	*Staphylococcus aureus*	*Alnus japonica* extracts at 0.1 mg ml^−1^	Quercetin influenced quorum sensing hence acted as an anti-biofilm compound against *S. aureus*	(97)
	Ten enteric bacteria and murine macrophage-like RAW264 cells	Quercetin dissolved in DMSO with final 25 μM in 1 ml of serum	Several flavonols like quercetin have a prebiotic-like effect on promoting *B. adolescentis* exerting anti-inflammatory activity by producing produces NO inhibitors	(81)
	Probiotic *Lactobacillus rhamnosus, Salmonella typhimurium*, and Caco-2 cells	Quercetin at a final concentration of 2 mg/ml	Quercetin could inhibit gut pathogen *Salmonella typhimurium* growth and adhesion to a human gut cell line, enhancing the probiotic *L. rhamnosus* proliferation, and adhesion	(82)
Kaempferol	Collagen-induced arthritis (CIA) model mice	Treating mice with kaempferol intragastrically (200 mg · kg^−1^ · d^−1^) and intraperitoneally (20 mg · kg^−1^ · d^−1^)	The high level of kaempferol produced distinct anti-arthritis effects in CIA model mice and regulated the intestinal flora and microbiotic metabolism	(98)
	*Helicobacter pylori* and human AGS cell ATCC® CRL-1739	The minimal inhibitory concentration (MIC) of kaempferol against *H. pylori* was 50 μM	Kaempferol suppressed *Helicobacter pylori* vacuolating cytotoxin A and cytotoxin-associated gene A translocation to AGS cells	(83)
	*Escherichia coli*	The MIC of kaempferol was 25 μg/ml	Kaempferol could have great anti-*Escherichia coli* activity and inhibition of DNA gyrase	(99)
Myricetin	—	—	The review summarized myricetin preclinical pharmacological activities, including antimicrobial properties with multiple mechanisms	(100)
	*S. aureus* infection *Galleria mellonella* model	Myricetin at 200 μM	Myricetin possessed the influence on several factors by *S. aureus*, including adhesion, biofilm formation, and staphyloxanthin production	(101)
**Flavones**				
Apigenin	—	—	The review summarized studies on antimicrobial effects of apigenin as well as the relationship between apigenin and human gut microbiota	(102)
	Human gut microbiota preparation from healthy female	Apigenin at 5, 12.5, 25, 50, and 100 μg/ml	Apigenin effectively inhibited the growth of both *Enterococcus caccae* and *Bacteroides galacturonicus* on a single strain; on the contrary, it enhanced the growth of *Enterococci* in the community	(103)
Baicalein	*Lactobacillus rhamnosus* JB3, human AGS cell ATCC® CRL-1739 and male C57BL/6 *Helicobacter pylori* infections mice	Baicalein at the concentrations of 1, 0.5, 0.25, 0.125, and 0.0625 mM	Baicalein and *Lactobacillus* spp. had a synergistic effect on eradicating *Helicobacter pylori* infections *in vitro* and *in vivo*	(84)
	SAMP8 mice	Administration baicalein with 200 mg kg^−1^ d^−1^	Baicalein altered the abundance of six genera in SAMP8 mice, reducing *Mucispirillum, Parabacteroides, Bacteroides*, and *Sutterella*, in contrast, increasing *Christensenellaceae*	(104)
	Male Wistar rats to establish high-fat, high-sugar diet (HFHSD) rat model	Baicalein at 50 mg kg^−1^ d^−1^	Baicalein could modulate the composition of gut microbiota via influence on the abundance of beneficial and pathogenic bacteria	(88)
Chrysin	Male CD Sprague-Dawley rats induced by fructose to establish metabolic syndrome model	Chrysin at 100 mg kg^−1^ d^−1^	Chrysin could affect fructose inducing rats intestinal microbiome, especially increasing *Firmicutes* to *Bacteroidetes* ratio	(105)
**Isoflavones**				
Genistein	HFD male C57BL/6J mice	HFD with 0.2% genistein approximately 3 mg kg^−1^ d^−1^	The abundance among the *Prevotella* and *Akkermansia* genera, particularly *Prevotella copri* and *Akkermansia muciniphila* under the treatment of genistein	(89)
	Female non-obese diabetic (NOD) mice	Oral administration of genistein at 20 mg kg^−1^ d^−1^	Perinatal genistein exposed NOD mice exhibited an increased level of *Enterobacteriales* that suggested a pro-inflammatory response	(91)
	Germ-free RAG2^−/−^ athymic female mice established breast cancer orthotopic xenografts	Feeding a special corn oil customized diet (genistein-0.25 g/Kg)	In the genistein-treated humanized mice, the abundance of genera *Lactococcus* and *Eubacterium* increased	(106)
**Flavan-3-ols**				
Epicatechin	——	——	This review introduced four main catechins found in green tea, including (–)-epicatechin (EC), (–)-epicatechin-3-gallate (ECG), (–)-epigallocatechin (EGC), and (–)-epigallocatechin-3-gallate (EGCG), had antimicrobial effects, such as damage to the cell membrane, inhibition of enzyme activity	(107)
	*Lactobacillus acidophilus* strains KCTC 3140, KCTC 3146, KCTC 3154, and KCTC3179	Epicatechin was quantified as 6.36 μg/ml in the *Bulnesia sarmienti* aqueous extracts	(–)-Epicatechin from *Bulnesia sarmienti aqueous* extract as the prebiotic active components enhanced the growth of four *Lactobacillus acidophilus* isolated from rat, pig, chicken, and human gut	(108)
Gallocatechin	Cystic Fibrosis patients fecal samples with ingesting flavonoid	——	Gallocatechin was found to be correlated with the family *Actinomycetaceae (Actinobacteria)*	(109)
**Flavanones**				
Naringenin	*Ruminococcus gauvreauii, Bifidobacterium catenulatum*, and *Enterococcus caccae*	Naringenin in a final concentration of 200, 150, 100, and 50 μg/ml in strain-specific broth containing 1% DMSO	Naringenin had the effect on the growth and genetic expression of three gut microbes, with the result of increasing *Bifidobacterium catenulatum*, inhibiting *Enterococcus caccae*, and no affection to *Ruminococcus gauvreauii*, both changing to the gene expression for all three strains	(110)
	*Escherichia coli* O157:H7 ATCC 43895 and *Vibrio harveyi* MM32	Naringenin at 6.25, 12.5, 25, 50, and 100 μg/ml	Naringenin had the potential to be a inhibitor of autoinducer-mediated cell–cell signaling for modulating the *Escherichia coli* biofilm and *Vibrio harveyi MM32* virulence	(111)
Eriodictyol	*S. aureus* USA300 strain ATCC BAA-1717 and human alveolar epithelial cell line (ATCC CCL185)	The MIC of eriodictyol against *S. aureus* was 512 μg/mL	Eriodictyol had the potential against *S. aureus* infection via downregulating alpha-hemolysin at the levels of expression and transcription	(112)
**Anthocyanins**				
Malvidin-3-glucoside	C57BL/6J male mice induced by DSS	The AIN-93M containing malvidin-3-glucoside at 24 mg kg^−1^ diet	Malvidin-3-glucoside reduced the abundance of pathogenic bacteria, such as *Ruminococcus gnavus*, and restored the *Firmicutes/Bacteroidetes* ratio	(113)
	Health human fecal samples	Malvidin-3-glucoside was inoculated at 20 mg/L and 200 mg/L	Malvidin-3-glucoside tested significantly enhancing the growth of *Bifidobacterium* spp. and *Lactobacillus-Enterococcus* spp.	(114)
Cyanidin-3-O-glucoside	Male Wistar rats induced by 3-chloro-1,2-propanediol	The diet with supplementation of 500 mg/kg cyanidin-3-O-glucoside	Cyanidin-3-O-glucoside was found to increase the relative abundance of *Lachnospiraceae* and *Actinobacteria* and might have beneficial regulating the communities of gut microbiota	(115)

### Effect of Gut Microbiota on the Bioaccessibility of Flavonoids

It is well-known that the bioaccessibility of flavonoids in the human gastrointestinal tract is induced by many factors (116), including its structure, hydrolyzing enzymes throughout the gastrointestinal tract, composition of the intestinal microbiota, and transporter proteins in intestinal epithelial cells. The gut microbiota contributes to further modification and transformation of flavonoids for beneficial physiological effects. Due to its specific and vast gene pool, the intestinal microbiota has great potential in exploring the metabolites of flavonoids, and catalyzing important reactions in the intestinal tract. The gut microbiota is thought to be helpful in various metabolic reactions, including biosynthesis, catabolism, conjugation, and modification by containing various types of enzymes (117). During the conversion of flavonoids, the catalysis reaction by gut microbiota includes the following three types; hydrolysis, cleavage, and reduction. These three types are further subdivided into O-de-glycosylation, ester hydrolysis, C-ring cleavage, de-lactonization, de-methylation, de-hydroxylation, and double bond reduction (17).

The most common flavonoids are in the form of glycosides, which require the removal of attached sugars before absorption. Besides carrying glucose, galactose, arabinose, rhamnose, and xylose in the form of rhamnosides or rutinosides also contribute to the structural diversity of flavonoids, such as hesperidin, naringin, narirutin, and neohesperidin. These compounds are needed to be hydrolyzed by enzymes either from gut microbiota or human intestinal enzymes before absorption. Some enzymes can cleave glycosidic linkages present in human cells, except rhamnosidases. It has been reported that some bacteria have β-glucosidase activities in the human intestinal microbiota, which mainly include *B. adolescentis, Bifidobacterium longum, Enterococcus faecalis, Bacteroides ovatus, Bacteroides uniformis, Parabacteroides distasonis*, and *Escherichia coli* (118–124), etc. The same is true for rhamnosidase (125–129). Recent advances have reported identifying human intestinal bacterial species and strains related to flavonoid conversion, which could catalyze de-glycosylation reactions, especially O-de-glycosylation (130). For example, 22 strains of *Bifidobacterium* representative among the eight major species from human origin were found to have the ability of bio-conversion of soy isoflavones (120). Tao et al. characterized and isolated intestinal bacteria from the human feces sample and investigated their ability to convert buddleoside by using UPLC-LTQ/Orbitrap/MS/MS, and found four strains showing more powerful conversion capability (128). After releasing their aglycones, the flavonoids can be metabolized by phase II biotransformation enzymes into glucuronidated, sulfated and methylated products (131), which then require specific transferases, including glucuronyl-transferase, phenol-sulfo-transferase, and acetyl-transferase. The catabolic reactions of flavonoids include the removal of methyl ethers, opening of carbon rings, and breaking of C-C bonds. These catabolic reactions are regulated by the enzymatic activities of anaerobic bacteria, such as *Clostridium* and *Coriobacteriaceae* (17). The phenolic rings of flavonoids often contain methoxy groups, and some strains have the ability to O-demethylate, such as *Bacterium Bautia sp. MRG-PMF1* and *Eubacterium limosum* (132–135). Recently, Feng et al. (136) have summarized that some intestinal bacteria could metabolize flavonoids in various metabolites with cleaved rings enzymes, mainly from *Enterococcus casseliflavus, Eubacterium ramulus, Clostridium orbiscindens sp. nov*. These microbe-derived metabolites were reported to possess multiple bioactivities in different metabolic pathways (136–138). Table 2 lists some main flavonoid-converting enzymes related to the human gut bacteria.

**Table 2:** Enzymes and microorganisms involved in the transformation of flavonoids by human gut bacteria.

**Reaction**	**Enzymes**	**Species/Strain**	**Major findings**	**References**
Deglycosylation	β-Glucosidase	*Bifidobacterium Adolescentis* *Bifidobacterium animalis subsp lactis* *Bifidobacterium bifidum* *Bifidobacterium breve* *Bifidobacterium catenulatum* *Bifidobacterium longum* *Bifidobacterium infantis* *Bifidobacterium Pseudocatenulatum*	Screened 22 strains of *Bifidobacterium* representative among eight major species from the human origin for their ability to bioconversion of soy isoflavones	(120)
			Screened five *Bifidobacteria* strains from the human origin for their specific beta-glucosidase activity and their metabolic competence in dietary flavonoids analyzed by high-performance liquid chromatography separations	(118)
			Selected two *Bifidobacterium* strains among 46 lactic acid bacteria for their relatively high beta-glucosidase activities with finding coding genes and successfully constructed several *bifidobacteria* expression vectors	(121)
		*Bifidobacterium lactis* *Lactobacillus plantarum* *Lactobacillus casei* *Lactobacillus acidophilus*	Investigated enzymatic potential of *Bifidobacteria* and *Lactobacillus* for converting delphinidin and malvidin glycosides and screened their β-glucosidase activity	(119)
		*Lactobacillus mucosae* INIA P50 *Lactococcus lactis* MG1363	The genes from *Lactobacillus mucosae* were cloned in *Lactococcus lactis* with special vectors, and their high beta-glucosidase activities and abilities to efficiently catalyze were shown	(124)
		*Lactobacillus casei* LP71 *Lactobacillus plantarum* E112 *Lactobacillus rhamnosus* E41 *Bifidobacterium pseudocatenulatum* C35	Fermented eight *lactobacilli* and two *bifidobacteria* strains and monitored their beta-glucosidase activities	(123)
		Lactic acid bacteria	Based on biochemical and genomic information, systematically summarized *lactic acid bacteria* having the glucosidase activities and the function of hydrolyzing plant metabolite glycoconjugates	(122)
	Rhamnosidase	*Escherichia sp*. 4 *Escherichia sp*. 34 *Enterococcus sp*. 45 *Bacillus sp*. 46	Characterized and isolated the intestinal bacteria from the fecal sample and investigated their conversion of bundle side using UPLC-LTQ/Orbitrap/MS/MS; as a result, four strains showed enzyme activities	(128)
		*Bifidobacterium longum* R0175 *Lactobacillus rhamnosus subsp. Rhamnosus* NCTC 10302	Investigated the ability of two probiotic bacteria to catabolise flavanones by HPLC-HR-MS	(129)
		*Bifidobacterium pseudocatenultum*	Investigated that *bifidobacteria* if could hydrolyze rutinosides by screening 33 strains and *Bifidobacterium pseudocatenulatum* showed the possibility in agreement with a putative alpha-l-rhamnosidase	(126)
		*Enterococcus avium* EFEL009	The strain was isolated and identified from the human fecal samples, and it showed enzymatic activities under anaerobic conditions	(127)
		*Bacillus sp*. 52 *Bacteroides sp*. 45, 42, 22 *Veillonella sp*. 32	Five human intestinal bacteria strains were found related to the deglycosylated route of rutin and showed α-l-rhamnosidase and β-d-glucosidase activities with using UPLC–Q-TOF/MS	(125)
Demethylation	—	*Bacterium Bautia sp* MRG-PMF1	Studied the capability of the human intestinal bacterium *MRG-PMF1* to the biotransformation and metabolizing of poylmethoxyflavones	(133)
			*MRG-PMF1* had the metabolic function to curcumin and other curcuminoids	(134)
			*MRG-PMF1* could biotransform poylmethoxyflavones to various demethylated metabolites	(135)
		*Eubacterium limosum*	The intestinal bacterium was used to test the capacity of O-demethylation and degradation of flavonoids	(132)
Ring cleavage	—	*Enterococcus casseliflavus* *Eubacterium ramulus* *Clostridium orbiscindens sp*. nov.	The review summarized the metabolism capabilities of different flavonoids to be the ring cleavage metabolites by the intestinal bacterial and their metabolic pathways	(136)
		*Bacterium* CG19-1	Newly isolated human intestinal bacterium CG19-1 from fecal suspensions was identified to convert puerarin	(137)
	—	*Eggerthella lenta* rK3 *Flavonifractor plautii* aK2	Isolated two bacterial strains from the human fecal suspension that were characterized to associate with the conversion of catechins	(138)
Double bond reduction	—	*Clostridium orbiscindens*	An anaerobic bacteria degrading quercetin isolated from human feces were identified by 16S rRNA gene sequence analysis and could transform several flavonoids under strictly anoxic conditions	(139)

## Gut Microbiota and IBD

**Figure 3:**
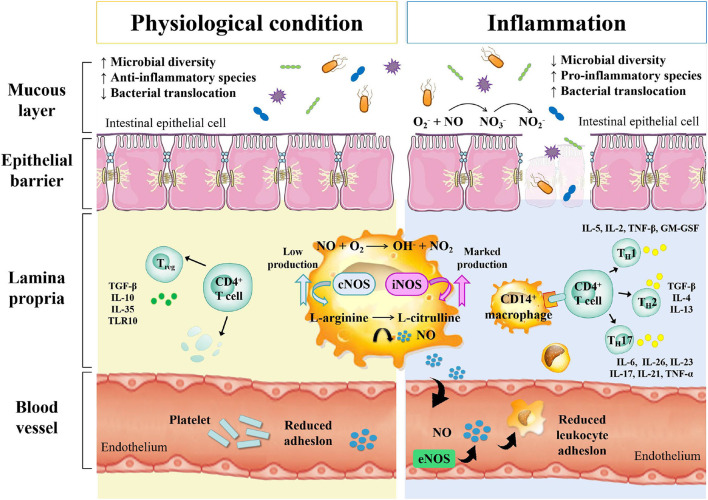
The relationship between gut microbe and IBD.

Inflammatory bowel disease is an autoimmune intestinal disorder, having UC and CD as the most common phenotype, characterized with the intermittent episodes having relapse and clinical remission, potentially causing intestinal injury and dysfunction. The UC is characterized with continuous inflammation, which is limited to the mucosa and extends from the rectum to a variable degree in the colon. The pathological features of UC are micro-abscesses, in which the neutrophils infiltrate into the lamina propria and intestinal crypts. Other histological feature includes the depletion of goblet cells. Unlike UC, the CD is characterized with the segmental and chronic inflammation and sharp demarcation between bowel segments, accompanied by the pathological features of transmural inflammation, granulomatous inflammation, and narrow or penetrating ulcers, involving any site of the gastrointestinal tract (140). The human gastrointestinal tract contains a complex and diverse microbial community, making a strong symbiotic relationship with the host. The gut homeostasis needs the interaction between commensal microbiota and host immune system (141). At present, the gut microbiota is reported to have multiple important functions, including the synthesis of essential vitamins, fermentation and digestion of other nutrients, and protection of the gastrointestinal from colonization by pathobionts. In general, the gut microbiota sustains a relative balance, which can be changed by environmental factors, such as anti-biotic exposure and diet (142–144). The gut microbiota is a key regulator of health and disease, which is, sometimes considered as an essential “organ” that provides nourishment, regulates the epithelial development, and instructs the innate immunity (145). However, it has also been linked to the risk of a variety of diseases, including the metabolic diseases, IBD, colorectal cancer, and allergic diseases (146). The excellent studies (Table 3), recently published by HMP and iHMP launched by NIH, have comprehensively characterized the metabolic changes in the microbiota and host immune responses during the IBD using multi-omics data, including macro-genomics, macro-transcriptomics, and macro-proteomics. All the relevant data is deposited in the Inflammatory Bowel Disease Multi-omics Database (IBDMDB) (3). This showed that the IBD, which is a heterogeneous disease, is caused by the genetic variabilities as well as the complex interactions between intrinsic host factors and environmental factors (147). However, the exact mechanism of pathogenesis of IBD is still remained to be elucidated, and there is no consensus theory according to existing literature. In the following, the complex interactions between IBD and gut microbiota are discussed to serve as primary targets for potential therapeutic strategies (Figure 3).

**Table 3:** The correlation between gut microbe and IBD in some research with various methods.

**Methodology**	**Study subject**	**Major findings**	**References**
Integrating taxonomic, metagenomic, metatranscriptomic, metaproteomic, and metabolic data	The Inflammatory Bowel Disease Multi'omics Database (IBDMDB) for 1,785 stool samples, 651 intestinal biopsies, and 529 quarterly blood samples	Demonstrate an increase in facultative anaerobes at the expense of obligate anaerobes, and molecular disruptions in microbial transcription (for example, among clostridia), metabolite pools (acylcarnitines, bile acids, and short-chain fatty acids), and levels of antibodies in host serum	(3, 10)
Healthy patient's biopsies	Five sites (cecum-ascending, transverse, descending, sigmoid, rectum) for healthy patient's biopsies	The composition of the microbiota in IBD patients differed from that of healthy controls. The high rate of bacterial DNA in the blood samples indicated translocation in inflammatory bowel disease	(148)
Whole-genome shotgun sequencing	Fecal DNA extracts from 13 healthy donors and 16 UC and 8 CD patients	*Enterococcus faecium* strains derived from UC patients displayed an inflammatory genotype that caused colitis	(149)
16S rRNA gene (RNA and DNA) pyrosequencing	Mucosal biopsies sampled from individuals of German, Lithuanian, and Indian origins	*Faecalibacteria* and *Papillibacter* belonged to *Clostridium leptum subgroup* had the respect to serving as reliable microbiomarkers	(150)
16S rRNA gene sequencing and Immunochip	Intestinal biopsies samples	Identified and confirmed a significant association between NOD2 risk allele count and increased relative abundance of *Enterobacteriaceae*, and 48 additional IBD-related SNPs had directionality of their associations with bacterial taxa	(151)
16S rRNA gene sequencing and microarray	Patients who had surgical management of UC and all patients had ileal pouch-anal anastomosis surgery at least 1 year prior to biopsy collection	Activation of host processes was inversely correlated with *Sutterella, Akkermansia, Bifidobacteria*, and *Roseburia* abundance and positively correlated with Escherichia abundance	(152)
16S rDNA sequencing and transcriptome analyses	Dextran sulfate sodium induced specific pathogen-free (SPF) and germ-free (GF) mice	Gut microbes were affected by diet and interfered with intestinal permeability and intestinal inflammation development	(153)
Metabolomics	The Human Metabolome Database (HMDB)	Established correlations between microbial composition and specific bacterial metabolic pathways; assessed the effects of small molecule products on IBD pathogenesis	(154)
Gut microorganisms	Two thousand three hundred and seventy-nine participants from two population-based cohorts (LLD and 500FG) and two disease cohorts (IBD and 300OB)	Identified several key species and pathways in IBD and obesity; provided evidence that altered microbial abundances in disease could influence their co-abundance relationship	(155)

### IBD and Microbial Dysbiosis

Studies have shown that cesarean section, non-breastfeeding, too clean life urbanization and anti-biotic abuse may lead to a lack of exposure to microorganisms in early life, resulting in the loss of adverse regulatory pathways, leading to an overactive immune response to the symbiotic gut microbiota. When the steady-state balance between the host and its microbial content is disrupted, it is called dysbiosis, which is considered to be closely related to IBD in some studies on animal models and humans. This uncontrolled intestinal immune response to the bacterial antigens leads to the activation of leukocytes and epithelial cells, causing the production of numerous cytokines and chemokines, thereby triggering a series of oxidation reactions and inflammatory responses (156). Differences in the compositions of intestinal microbiota exist between IBD patients and healthy people. Similarly, Rehman et al. profiled the bacterial community by analyzing 89 mucosal biopsies samples from German, Lithuanian, and Indian individuals and found that the *Faecalibacteria* and *Papillibacter*, which belong to the *Clostridium leptum subgroup*, served as reliable microbiota-based biomarkers (150). The relative abundances of *Firmicutes* and *Enterobacteriaceae* were also found to be correlated with the IBD (157). Recent metagenomic analyses also revealed the effects of the gut microbiota's dysbiosis at the strain level, demonstrating *Enterococcus faecalis* as an inflammatory genotype from UC patients (149). Moreover, the association between *Malassezia* and CD has also been demonstrated in the dextran sulfate sodium (DSS) -induced mouse models (158). *M. restricta* mainly triggers innate inflammation through CARD9 and is recognized by anti-fungal anti-bodies in patients with CD; it will produce strong inflammatory cytokines from innate cells carrying IBD-related CARD9 polymorphisms and aggravate colitis in mice model. Among various chemically induced colitis models, the DSS-induced colitis model is widely used because of its simplicity and many similarities with human UC. The acute, chronic, and recurring models of intestinal inflammation can be achieved by modifying the concentration and frequency of DSS.

### IBD and Immune Response

The host immune system and indigenous gut microbiota co-evolve with each other, maintaining a rigid balance between tolerance to the symbiotic bacteria and responding to the threat from pathogenic bacteria (159). The balance of T_reg_/T_H17_ with pro-inflammatory and anti-inflammatory cytokines is essential for efficient host gut homeostasis and is directly affected by the gut microbiota, which has been demonstrated in mouse models (160, 161). Under the transcriptional regulation of retinoic acid-related orphan receptor (ROR)-γt, the T_H17_ mediates effector functions, which is a characteristic feature for the production of signature cytokines, including interleukin (IL)-17, IL-21, IL-22, IL-23, and IL-25, which, in turn, induce other pro-inflammatory cytokines and chemokines, promoting tissue inflammation. The T_reg_ cells are regulated by the transcription factor forkhead box P3 (Foxp3), and can be divided into natural and induced T_reg_ cells (162). The germ-free mouse models showing the symptoms of intestinal immune deficits demonstrated that the microbiota was required for the development of intestinal immune system (163). The increase in the intestinal T_H17_ and T_H2_ cells and decrease in the ROR γt Treg cells were reported in the germ-free mice being supplemented with the IBD microbiota (164). Some species in the gut microbiota directly or indirectly participate in the inflammatory processes. Their pathogenetic effect is based on the induction of proinflammatory immune cells, followed by the effector function. A number of studies have identified several specific commensal bacteria with intestinal T_reg_-inducing capacity, such as *Bacteroides fragilis* and *Clostridium* (163, 165, 166). Interestingly, a study found the commensal bacterium, involved in the pathogenesis of CD, produced the anti-inflammatory proteins, revealing its anti-inflammatory effect by inhibiting the NF-κB pathway in the dinitrobenzene sulfonic acid (DNBS)-induced colitis mouse models (167). On the other hand, a study found that the total mucosa-associated bacteria increased in the UC and CD samples from the normal intestinal epithelium, while the quantity and abundance of *Akkermansia muciniphila* reduced by many fold (168). Another study found that the human commensal bacterial strain, *B. fragilis*, had a protective effect in DSS-induced mouse models of experimental colitis (169).

### IBD and Barrier Disruption

The alterations in the gut microbiota resulted from the increase in the nitrosative and oxidative stress in the intestinal environment during IBD include the decrease in microbial diversity and the increase in the relative abundance of facultative anaerobe (7). One of the typical symptoms of IBD pathology is the dysfunction of intestinal barrier with increasing intestinal permeability, which results from disruption of tight junction proteins and release of pro-inflammatory mediators, including ROS and RNS (170). As a result, the aberrant mucosal immune response affects the genetically predisposed individuals, causing the pathologic alterations in their intestinal microbiota (171). In healthy people, the normal intestinal microbiota inhabiting the colon mucus is relatively stable, which does not trigger the inflammatory response. This is due to the inner and outer mucus layers of colon, which are consisted of mucins, trefoil peptides, immunoglobulins and other proteins, consequently maintaining the barrier integrity (172). The anti-microbial proteins also play an important role in the prevention of the overgrowth of pathogenic strains (173). The dysregulation of mucosal immune system usually results in the pathogenic immune response to the intestinal commensal microbiota (174). Vrakas et al. collected blood and tissue biopsy samples from the active/inactive UC and CD adult patients, as well as healthy individuals, and determined their composition of gut microbiota using real-time quantitative reverse transcription PCR. The result showed that in comparison with healthy controls, the total bacterial DNA concentration levels were higher in the IBD patients with the increase in *Bacteroides* spp. and reduction in *Clostridium leptum* group and *Faecalibacterium prausnitzi* (148).

## Flavonoids and IBD

**Figure 4:**
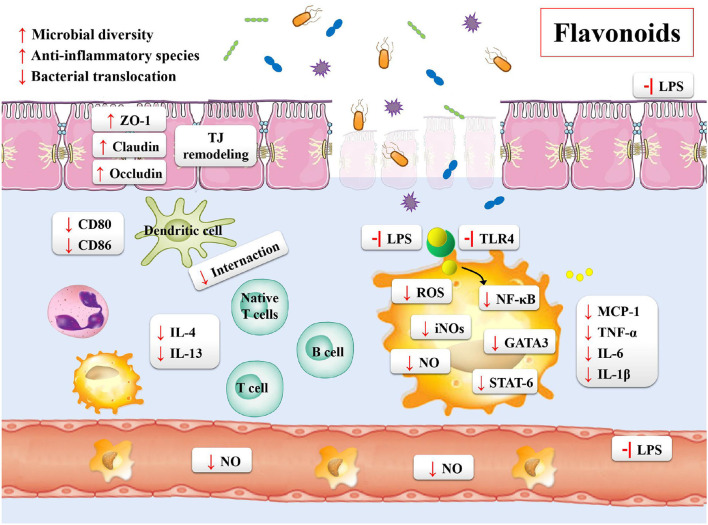
The potential mechanisms of flavonoids on gut microbiota and IBD.

Inflammatory bowel disease is characterized by the chronic inflammation of gastrointestinal tract. The development of disease course seriously impairs the quality of life of the patients, who then require sustained drug treatments and surgical interventions. Based on these situations, the treatment targets of IBD is the remission of symptoms during the acute flare and lowering the chronic inflammatory state (20). In order to overcome these problems, the effective and safe treatment strategies for the prevention and treatment of IBD are needed. Flavonoids may be one of such potential agents (Table 4). Previous studies have shown the following beneficial effects of flavonoids on the gastrointestinal tract: maintaining the integrity of the intestinal barrier; preventing the intestinal wall from pharmacological insults and food toxins; regulating the gut immune system; shaping the profiles of microbiota; regulating the gut hormonal secretions by enteroendocrine cells (Figure 4) (187).

**Table 4:** The affection of flavonoids on gut microbiota and IBD.

**Compound**	**Animal model**	**Dosage form**	**Major findings**	**References**
Naringenin	DSS-induced colitis male BALB/c mice	Feeding a diet containing 0.3% (wt:wt) naringenin	Feeding naringenin attenuated the increased DAI and colon shortening and tended to suppress the increased cytokine expression	(175)
Sinensetin	TNBS and DSS induced colitis male Sprague-Dawley rats	Sinensetin at low-dose 20 mg/kg, or high-dose 80 mg/kg	Sinensetin reversed the colitis-associated increase in intestinal permeability, significantly promoted epithelial cell autophagy, further decreased apoptosis, and reduced mucosal claudin-2	(176)
Kaempferol	Coculture model of intestinal epithelial cells and intestinal microvascular endothelial cells	Kaempferol at 80 μM	Kaempferol alleviated the IL-8 secretion, and barrier dysfunction of the Caco-2 monolayer, had a protective effect against barrier dysfunction via preventing the activation of the NF-κB signaling pathway	(177)
	DSS-induced colitis male C57BL/6 mice	Rape bee pollen extracts at 21.2 and 10.6 g/kg (containing kaempferol 19.87 mg/g)	The kaempferol in rape bee pollen extracts altered the gut microbial structure of colitis mice, significantly reduced the abundances of *Allobaculum* and *Bacteroides* and markedly increased the abundance of *Lactobacillus*	(178)
	Female C57BL/6J mice	0.1 and 0.3% kaempferol diets	Kaempferol decreased plasma levels of NO and PGE2, suppressed colonic mucosa MPO activity, and up-regulated goblet cell function marker TFF3 mRNA	(179)
Baicalin	TNBS-induced colitis Sprague-Dawley rats	Baicalin at 100 mg kg^−1^ d^−1^, per rat by gastric lavage	Baicalin alleviated TNBS-induced colitis by reducing the release of IL-6, TNF-α, and IL-1β and increasing the level of IL-10, promoting the expression of tight-junction proteins ZO-1 and β-catenin	(180)
	DSS induced colitis C57BL/6J male mice	Baicalin at 20 mg/kg	The target cells of activated T cells in the gut, STAT4 transcription in CECs was downregulated by baicalin	(181)
Astragalin	DSS-induced acute murine colitis model	Oral gavage astragalin at 2 and 5 mg/kg	Astragalin reduced the level of phosphorylated IkappaBalpha and decreased the production of the inflammatory cytokines IL-6, IL-8, and TNF-alpha	(182)
	DSS-induced colitis male C57BL/6 mice	Daily oral doses of 200 μL of astragalin at 50, 75, 100 mg/kg	Astragalin might exert a good anti-UC effect through microbiota/LPS/TLR4/NF-kB-related pathways in mice	(183)
Quercetin	Pathogen-free female C57BL/6 mice infected by *Citrobacter rodentium*	Receiving a basal rodent diet supplemented with 30 mg/kg quercetin	The *Citrobacter rodentium*-induced colitis mouse model proved quercetin supplementation could enhance the populations of *Bacteroides, Bifidobacterium, Lactobacillus, Clostridia*, and significantly reduced those of *Fusobacterium* and *Enterococcus*	(184)
	Adoptive T-cell transfer model of chronic colitis in Rag1^−/−^ mice	Orally receiving 10 mg/kg quercetin once every 3 days	Quercetin administration lessened the enteric bacteria flora, increased the *Bacteriodetes/Firmicutes* ratio with a proportional decrease in the Gram-negative *Proteobacteria* and Gram-positive *Actinobacteria*, increased the *Bacteroides*, decreased segmented filamentous bacteria groups and *Escherichia coli*	(185)
	Male Wistar rats	Feeding standard diet and quercetin (150 mg/kg)	According to the result of Illumina MiSeq sequencing and Illumina HiSeq, quercetin combined with alliin could reshape the gut microbiota composition and alter the immunologic function of colonic epithelial cells	(186)

### Flavonoids: Mucosal Integrity and the Function of Intestinal Epithelial Barrier

The mucosal barrier is imperative for the maintenance of intestinal homeostasis. The dysfunction of intestinal barrier is correlated with the etiology of IBD (188). One of the prospective studies about the IBD patients and mucosal healing demonstrated the relationship between impaired intestinal permeability and ongoing bowel symptoms of diarrhea or abdominal pain (189). The *in vivo* DSS-induced mouse colitis models also demonstrated that the increase in the gut permeability resulted in chronic inflammatory responses (190, 191). The two main factors used for the evaluation of the function of intestinal mucosal barrier include the integrity of mucus layer and the production and assembly of tight junction proteins (192). The mucus layer provides a physicochemical barrier to protect the surface of epithelial cells. The tight junction proteins are the main determinants of intestinal physical barrier. The expression and association of the tight junction proteins and actin cytoskeleton determine the permeability of tight junction proteins, which are dynamically regulated by various intracellular signaling molecules. Studies have reported that flavonoids could enhance the integrity of tight junction proteins, including ZO-2, occludins, and occluding. Quercetin, myricetin, and kaempferol could enhance tight junction protein expression and inhibit PKCδ in human Caco-2 cells (193, 194). In addition, the DSS-induced mouse model has also demonstrated that the supplement of naringenin decreased the disease activity index and expression of inflammatory cytokine (IL-6 and IL-17A) (175). In similar animal models, the sinensetin reversed the colitis-associated increase in the intestinal permeability, promoted the autophagy of epithelial cells, decreased the apoptosis of epithelial cells, and reduced the mucosal claudin-2 (176).

Many studies currently focus on the association of morphological and functional changes in the vascular endothelium with IBD (177, 195–197). The functional and structural changes in vascular endothelium lead to the activation of endothelial cells. These changes cause the expression of a variety of cell-adhesion molecules and chemokines on the endothelia cell surface, including ICAM-1, VCAM-1, and IL-1β, and the enrichment of leukocyte activation following inflammatory responses. As compared to healthy controls, the IBD patients showed an increase in the adhesion of CAM and leukocyte (195). Microvascular expression of CAMs could mediate the recruitment of circulating leukocytes. The quercetin has been reported to reduce the overproduction of TNF-α, IL-1β, interleukin-6, ICAM-1, and VCAM-1 in the cellular model of lipopolysaccharide (LPS)-induced rat intestinal microvascular endothelial cells (196). Using the same *in vitro* models, investigating the integrity of intestinal endothelial barrier, the naringin effectively ameliorated the disruption of gut-vascular barrier (197). The kaempferol showed similar effects. Moreover, it has a protective effect against the dysfunction of barrier via preventing the activation of NF-κB signaling pathway in the LPS-induced epithelial-endothelial co-culture model (177).

### Flavonoids and Immunomodulation

The different activities and properties of flavonoids have important implications in the activation, maturation and signal transduction of cells and the production and secretion of several cytokines in immune cells (198). A recent study showed that the astragalin reduced the level of pro-inflammatory cytokines and their mRNAs, such as TNF-α, IL-6, and IL-1β, and affected the key relative groups abundance of potentially beneficial *Ruminococcaceae* and potentially harmful *Escherichia-Shigella* (183). As mentioned previously, the imbalance of the immune system is associated with the pathogenesis of IBD. The adaptive immune system is usually considered as a main contributor to the pathogenesis of IBD, which may arise from an increase in the pro-inflammatory factors driven by TH cells and ineffectual anti-inflammatory T_reg_ cells (199). For example, using the DSS-induced mouse colitis models, Tao et al. found that the natural icariin inhibited the T_H1_/T_H17_ responses by suppressing the activation of STAT1 and STAT3, leading to the mitigation of inflammation (200). Abron et al. demonstrated that the genistein skewed the M1 macrophages toward M2 phenotypes, and reduced the systemic cytokine levels in part to attenuate the colitis symptoms (201). The majority of published studies performed using the experimental models of colitis have indicated that the altered immune response was correlated with the increasing release of pro-inflammatory cytokines, including IFN γ, TNFα, IL-6, IL-1β, GM-CSF, and IL-17A (20). With the increase in the mucosal permeability, the Toll-like receptors (TLRs) in endothelial cells of the intestinal epithelium are activated by the LPS exposed on the surface of gram-negative bacteria. In such case, the quercetin was found to prevent activation of inflammatory responses by inhibiting the LPS-induced expression of TLR4 in endothelium, through the MyD88-dependent NF-κB, MAPKs, and STAT pathways (202). In the 2,4,6-trinitrobenzenesulfonic acid (TNBS)-induced colitis mouse models, the baicalin exhibited anti-inflammatory effects by inhibiting the activation of TLR4/NF-κB and PI3K/AKT pathways (180, 203). Likewise, the same *in vivo* models showed the beneficial effect of oral tangeretin, alleviating the disease symptoms in TNBS-induced colitis models. The possible mechanisms of action of tangeretin might be the inhibition of LPS binding to the immune cells, such as dendritic cells, by suppressing the expression of IL-12 and TNF-α as well as the activation of NF-κB (204). The similar results were also observed for astragalin and taxifolin (182, 205).

### Flavonoids and Gut Microbiota

The interaction between flavonoids and microbiota offers considerable potential for the maintenance of gastrointestinal and systemic health. The beneficial effects of flavonoids for improving the health are not only attributed to their direct impact on the colon, but also to their alteration of the intestinal microbiota and performance of metabolism, which provide a therapeutic basis for the IBD with dysbiosis (187). Many studies have reported that the commonly observed variations in the IBD samples mainly focused on the reduced diversity of gut microbiota with reduction in the relative abundance of reducing *Firmicutes* and increasing *Proteobacteria* (206).

Flavonoids are reported to positively regulate the composition of gut microbiota. Several studies have shown a great therapeutic potential of the quercetin for the IBD patients, suppressing the abnormal expression of proinflammatory cytokines and modifying the gut microbiota by increasing the relative abundance of *Bacteroides, Bifidobacterium, Lactobacillus*, and *Clostridia*, while reducing the relative abundance of *Fusobacterium* and *Enterococcus* (184, 185). In addition, both the quercetin aglycone alone and in combination with the mono-glycosides exhibited higher Chao1 and Shannon indices and a lower Simpson index to counteract the adverse effect by colitis (207). Furthermore, the flavonoids upregulated the beneficial bacteria and down-regulated the growth of pathogen. Recently, the *A. muciniphila* showed protective effects against colitis in the DSS-induced colitis mouse models (208), confirming its main biological functions, which included the maintenance of gut barrier function and host metabolism (168, 209). Dietary supplementations with some flavonoids positively regulates this bacterial growth (79, 210, 211). For example, cranberry extract treatment markedly increased the proportion of *A. muciniphila*, and it was identified that the daily intake of flavonols were at 18.8 mg kg^−1^ d^−1^, total anthocyanins were at 6.6 mg kg^−1^ d^−1^ (79). The *Clostridium difficile* infection (CDI) has dramatically increased over the past decade worldwide (212). This bacterium induces aberrant inflammatory immune responses, which causes the breakdown of gastrointestinal barrier in the genetically susceptible individuals, leading to the development of IBD (213). The literature evidence suggests that the flavonoids significantly reduce toxin synthesis, sporulation, and spore outgrowth of *C. difficile*, while down-regulates the genes critical for pathogenesis (214, 215).

The colonic inflammation stimulates the production of IFN-γ, generating ROS by phagocytic innate immune cells, which causes a series of radicals as their end-products by facultative anaerobes. As a result, the bacterial diversity is decreased. Nevertheless, the beneficial effects of flavonoids for promoting the gut health are due to its anti-oxidant and free radical-scavenging activity (216). Most of the flavonoids assayed have the hallmark features of possessing numerous hydroxyl groups with the ability of amelioration oxidative stress (20, 216). A number of studies have reported that the flavonoids inhibit the expression of iNOS, while reduce the production of NO (20, 217). Moreover, the flavonoids enhance the activities of different enzymes having anti-oxidant properties, such as glutathione, superoxide dismutase and gluthathione peroxidases (218, 219).

## Conclusions and Future Perspectives

In this review, the flavonoids have been systematically summarized, providing evidences with a focus on the interactions between flavonoids and gut microbiota and their potential therapeutic effect, including their potential mechanisms of action, on IBD. However, it should be noted that there are still many problems need to be solved. Currently, some aspects of the pathogenesis of IBD are not completely understood. The gut microbiota has been widely recognized as a key factor for the intestinal homeostasis and has correlation with the pathogenesis of IBD (220). The current therapies for IBD are comprised of corticosteroids, immunosuppressants, anti-biotics, and biological agents. However, these therapies are neither curative nor cost-effective. Furthermore, these therapies do not target the microorganisms directly for the cause or contribution to inflammation (221). Thus, to develop more effective strategies for the treatment of IBD still remains a challenge. The flavonoids have received much attention for their biological properties, as well as their anti-oxidant and anti-inflammatory activities have been well-documented. Meanwhile, the flavonoids and their metabolites also affect the intestinal ecology by regulating the microbiota with bacteriostatic or bactericidal effects for the pathogenic and harmful bacteria, and prebiotic effects for the beneficial bacteria (222). On the other hand, due to the low bioavailability of flavonoids, 90% of them persists in the colon. The gut microbiota exhibits a great metabolic capacity of metabolizing the flavonoids via hydrolyzation, de-methylation, de-hydroxylation, and de-carboxylation, resulting in smaller metabolites, which are absorbed across the intestinal mucosa to benefit human health. In summary, flavonoids have tremendous therapeutic potential for the treatment of IBD. However, most flavonoids have poor water solubility, so their clinical application is hindered. Future studies could focus on IBD treatment by targeting gut microbiota and studying flavonoid drug delivery methods, including proteins, peptides, monoclonal anti-bodies, nucleic acids, and living cells, etc. The potential molecular mechanisms or toxicological assessments of flavonoid regulation of intestinal microbiota have not been well-elucidated, nor have their actual efficacy in the treatment of human IBD been proven. Thus, further research should also pay attention to more animal or cellular models and even clinical data to evaluate the safety and efficacy of flavonoid monomer molecules targeting gut microbiota against IBD.

## Author Contributions

HH conceived and designed the review. LW was mainly responsible for writing articles. MG and GK revised the manuscript. All authors were responsible for reading and approving the manuscript.

## Funding

The present study was supported by grants from National Key Research and Development Project (No. 2019YFA0905600), Science and Technology Program of Tianjin, China (No. 19YFSLQY00110).

## Conflict of Interest

The authors declare that the research was conducted in the absence of any commercial or financial relationships that could be construed as a potential conflict of interest.

## Publisher's Note

All claims expressed in this article are solely those of the authors and do not necessarily represent those of their affiliated organizations, or those of the publisher, the editors and the reviewers. Any product that may be evaluated in this article, or claim that may be made by its manufacturer, is not guaranteed or endorsed by the publisher.

## Abbreviations

IBD: inflammatory bowel diseases
UC: ulcerative colitis
CD: Crohn's disease
MetaHIT: metagenomics of human intestinal tract
NIH: National Institutes of Health
HMP: human microbiome project
iHMP: integrative human microbiome project
NO: nitric oxide
AGS: human gastric adenocarcinoma cell line
HFD: high-fat diet
NOD: non-obese diabetic
QS: quorum sensing
AHL: acyl homoserine lactone
UPLC-LTQ/Orbitrap/MS/MS: ultra-high pressure liquid chromatography with a linear ion trap-high resolution Orbitrap mass spectrometry system
IBDMDB: inflammatory bowel disease multi-omics database
DSS: dextran sulfate sodium
Treg: regulatory T cells
TH: T helper cells
ROR: retinoic acid-related orphan receptor
IL: interleukin
Foxp3: Forkhead box P3
DNBS: dinitrobenzene sulfonic acid
ROS: reactive oxygen species
RNS: reactive nitrogen species
PCR: polymerase chain reaction
LPS: lipopolysaccharide
TLRs: Toll-like receptors
TNBS: 2,4,6-trinitrobenzenesulfonic acid
CDI: *Clostridium difficile* infection
iNOS: inducible nitric oxide synthase.
